# Coronary calcification and bone microarchitecture by high-resolution peripheral quantitative computed tomography from the São Paulo Ageing and Health (SPAH) Study

**DOI:** 10.1038/s41598-022-08839-0

**Published:** 2022-03-28

**Authors:** Luis Fernando Escobar Guzman, Neuza Helena Moreira Lopes, Georgea H. Fernandes Torres, Liliam Takayama, Solange de Sousa Andrade, José Ramón Lanz-Luces, Rosa Maria R. Pereira, Carlos Eduardo Rochitte

**Affiliations:** 1 Unidade Clínica de Cardiogeriatria, Instituto do Coracao (InCor), Hospital das Clinicas HCFMUSP, Faculdade de Medicina, Universidade de Sao Paulo, Av. Dr. Enéas C. de Aguiar 44, São Paulo, SP 05403-900 Brazil; 2 Serviço de Henodinâmica e Cardiologia Intervencionista, Instituto do Coracao (InCor), Hospital das Clinicas HCFMUSP, Faculdade de Medicina, Universidade de Sao Paulo, Sao Paulo, SP Brazil; 3 Serviço de Reumatologia, Hospital Universitário Alcides Carneiro, Universidade Federal de Campina Grande, Paraíba, Brazil; 4 Instituto Paulista de Doenças Cardiovasculares, Sao Paulo, SP Brazil; 5 Laboratório de Metabolismo Ósseo, Serviço de Reumatologia, Hospital das Clinicas HCFMUSP, Faculdade de Medicina, Universidade de Sao Paulo, Sao Paulo, SP Brazil; 6 Serviço de Ressonância Magnética e Tomografia Cardiovascular, Instituto do Coração (InCor), Hospital das Clinicas HCFMUSP, Faculdade de Medicina, Universidade de Sao Paulo, São Paulo, SP Brazil

**Keywords:** Cardiology, Rheumatology

## Abstract

Epidemiological studies reveal a link between osteoporosis and the risk of ischemic cardiovascular disease. We illustrate an association between coronary calcification and bone microarchitecture in older adults based on the SPAH study. This cross-sectional research comprised 256 individuals subjected to cardiac coronary computed tomography angiography (CCTA) for coronary artery calcification (CAC), high-resolution peripheral quantitative computed tomography (HR-pQCT) at the tibia and radius with standardized *z* score parameters, and dual-energy X-ray absorptiometry (DXA) to evaluate bone status. We used Student’s *t* test and the Mann–Whitney and Chi-squared tests for comparison of basal measurements. Association analysis was performed using the Poisson regression model with adjustment for CAC and sex. Multivariate analysis revealed different bone variables for predicting CAC in DXA and HR-pQCT scenarios. Although most of the bone parameters are related to vascular calcification, only cortical porosity (Ct.Po) remained uniform by HR-pQCT. Results for were as follows: the tibia—women (exp β = 1.12 (95% CI 1.10–1.13, p < 0.001) and men (exp β = 1.44, 95% CI 1.42–1.46, p < 0.001); the radius—women (exp β = 1.07 (95% CI 1.07–1.08, p < 0.001) and men (exp β = 1.33 (95% CI 1.30–1.37, p < 0.001). These findings suggest an inverse relationship between CAC and cortical bone content, as assessed by HR-pQCT, with higher coronary calcification in individuals older than 65 years.

## Introduction

Atherosclerotic disease and osteoporosis are highly prevalent pathologies in older adults^1, 2^. In addition to sharing risk factors^3^, evidence suggests that the diseases have common pathophysiological mechanisms^4–6^.

Furthermore, bone loss is associated with cardiovascular disease severity at the time of diagnosis, and the degree of vascular calcification is a significant predictor of bone loss and vertebral and nonvertebral fractures^7, 8^.

In this scenario, coronary artery calcification (CAC), as calculated by the Agatston score^9^, has proven to be a powerful tool in personalized cardiovascular event risk assessment^10^. Its negative predictive value is reported to be 87% using a cutoff point of 100^11^. Additionally, the score is an independent predictor of cardiac outcomes up to 10 years, better than other combined risk factors that individualize atherosclerotic burden in a clinical form (ethnicity, age, and sex)^12^^.^

Areal bone mineral density (aBMD) assessed by dual-energy X-ray absorptiometry (DXA) is the most used exam for osteoporosis diagnosis and fracture risk assessment. It is a reproducible method with a low radiation dose. The main parameter is the *T*-score, which is considered normal when greater than − 1.0 standard deviation (SD); osteopenia is considered at − 1 to − 2.5 SD and osteoporosis at less than − 2.5 SD^13^. A reduction in aBMD has been independently associated with a higher number and greater severity of vertebral and hip fractures^13, 14^. Nevertheless, the Rotterdam study^15^ revealed a need for a more predictive tool based on results indicating that more than 60% of fractures occur in individuals considered to have a low risk. With its routine use worldwide, some authors have specified key limitations with regard to pitfalls in measuring and interpreting bone mineral density (BMD)^16^. For example, the need for internal rotation of the femur in 15–20% of cases favors an anteverted position, with a further 2.8% increase in BMD^17^. Other authors recommend avoiding Ward’s area^18, 19^, in which osteoporosis can be overestimated^20^.

High-resolution peripheral quantitative computed tomography (HR-pQCT) is a method that can address the deficiencies of DXA. It allows 3-D evaluation of trabecular and cortical bone compartments separately, as well as volumetric bone mineral density and structural parameters. In addition, HR-pQCT provides data on biomechanical characteristics through finite element software^21, 22^, offering better predictors of fracture risk than DXA^23^.

To the best of our knowledge, only one study has shown an inverse association between trabecular bone volume (measured with HR-pQCT) and CACs, though only end-stage dialysis chronic kidney disease (CKD) patients were included^24^. Hence, the relationship in asymptomatic individuals with subclinical pathologies according to HR-pQCT remains unclearknown using HR-pQCT. This study aimed to investigate whether there is an association between coronary artery calcification and density and BMD (obtained by HR-pQCT) in a cohort of community-dwelling older adults.

## Materials and methods

### Population of study

**Figure 1:**
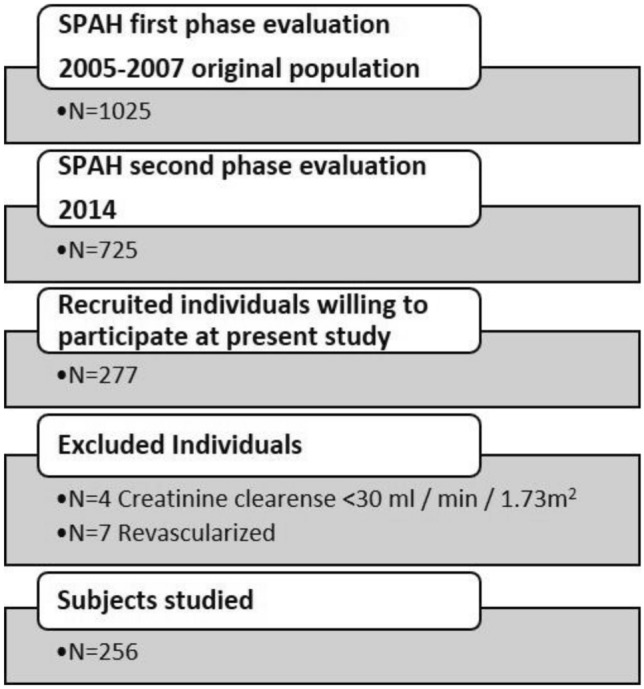
Flow diagram of the study population.

This was a cross-sectional study of 256 individuals older than 65 years. The data belonged to a third party (a cohort of community-dwelling older adults living in São Paulo city and participating in the São Paulo Ageing and Health Study [SPAH] study)^25^ (Fig. 1). Contact with the participants was initially achieved by phone, after which a physician explained the study aims and logistics. Recruitment was carried out from 4/30/2014 to 4/13/2015. Exclusion criteria were individuals with a history of liver failure, creatinine clearance (CrCl) < 30 mL/min/1.73 m^2^, hyperthyroidism, hypothyroidism, uncontrolled diabetes, malabsorption, other bone metabolic diseases (primary hyperparathyroidism, osteomalacia) based on previous laboratory results concerning bone metabolism, surgical myocardial revascularization, percutaneous coronary intervention, and chronic steroid use. The research was approved by the Ethics Committee of the University of São Paulo School of Medicine (CAPPesq # 522.369), as based on the World Medical Association’s guidance and principles, and subject to the Declaration of Helsinki, including its last amendment by the General Assembly, Fortaleza, Brazil, October 2013^26^.

All participants agreed to and signed the written informed consent form prior to enrollment into the study, thereafter, they were asked to fill out a questionnaire, which captured personal pathological background, current medications, and health behaviors. Specially trained examiners performed anthropometric measurements. Height and weight were measured using a digital scale with a stadiometer (Mettler Toledo, mod. 2096PP, Toledo do Brasil Indústria de Balanças Ltda); all measurements were taken closest to 0.1 kg and 0.1 cm, and each participant’s weight in kilograms was divided by the square of their height in meters to calculate body mass index (BMI). Waist circumference measurement was also performed.

Systemic arterial hypertension (SHT) was defined as documented history or treatment with antihypertensive medications; diabetes mellitus (DM) was defined as physician diagnosis and/or use of insulin or oral hypoglycemic agents and dyslipidemia (DLP) as a known but untreated disease or by current use of hypolipidemic drugs. Metabolic syndrome was defined using the recommendations of the third NCEP panel report^27^. A positive smoking history was considered current or previous smoking. A history of acute myocardial infarction (AMI), angina, stroke, carotid disease, osteoporosis, and coffee consumption was self-reported. The degree of physical activity was classified as either low (no housework), moderate (housework, gardening, nonregular walking), or high (regular physical activity outside the usual routine, e.g., walking or dancing at least twice a week for 30 min^28^). Coffee consumption data collection was based on a previously validated questionnaire^29^: participants were asked if they consumed at least one cup daily (50 mL = 1 cup). Alcohol intake was split into four groups: current drinker, intake in the last 3–4 years, never, or in a social manner. Bone *T*-score (BT score) ≤ − 2.5 was considered the smallest measurement acquired by DXA for the lumbar spine, total hip, or femoral neck. New fracture was self-reported and considered if present in the last 5 years after the initial entry-study date. To calculate creatinine clearance (CrCl), we used the Chronic Kidney Disease Epidemiology Collaboration (CKD-EPI) equation^30^.

### Laboratory parameters

Blood samples were collected to assess the lipid profile, creatinine, glycemia, insulin, and biochemical bone metabolism profile (calcium, phosphorus, alkaline phosphatase, PTH, and 25-hydroxyvitamin D) and perform ultrasensitive PCR.

Bone remodeling markers were analyzed in 10 mL of fasting blood stored at − 80 °C. The following markers of serum bone metabolism were examined: CTX (C-terminal telopeptide type I collagen) and P1NP (aminoterminal propeptide of type I procollagen) by electrochemiluminescence (Elecsys systems, Roche Diagnostics, Mannheim, Germany). The intra-assay coefficients of variation (CVs) for CTX and P1NP were 2.5 and 2.2%, respectively.

### Evaluation of coronary calcification

Image acquisition for coronary calcium evaluation was performed using a 320-detector CT system (Aquilion One ViSION, Toshiba Medical System, Japan), with cardiac synchronization using prospective triggering with a maximum temporal resolution of 125–250 ms as referenced in the literature^31^. The produced slice thickness of 0.5 mm was reconstructed to a 3 mm slice thickness for analysis, X-ray tube peak voltage was fixed at 120 kV, and the tube current was adjusted to the patient's body size, with a range of 250–450 mA. The adaptive iterative dose reduction 3-dimensional algorithm (AIDR 3D) was used for iterative reconstruction^32^, resulting in a radiation dose ranging from 2.6 to 4.0 mSv. CAC values were calculated using the Agatston score^9^ at a dedicated workstation (Aquarius workstation, TeraRecon, Inc, San Mateo, CA). The nonenhanced scan protocol was used to analyze the calcium score evaluation. The images were evaluated by one specialist medical doctor with at least 2 years of specific training who was unaware of the patient’s clinical and tomographic information.

### Evaluation of the distal tibia and radius using HR-pQCT

The distal segment of the tibia and radius were evaluated. The measurement included 110 slices, corresponding to a 9.02 mm section along the axial direction. For the distal radius and tibia, the first slice of the scan was positioned at fixed distances from the reference line of 9.5 and 22.5 mm, respectively^33^. Microarchitecture was measured using a three-dimensional HR-pQCT system (Xtreme CT; Scanco Medical AG). This system allowed simultaneous acquisition of a stack of parallel slices with a resolution of 82 µm (voxel size). The following imaging parameters were used: 60 kVp effective energy, 95 mA X-ray tube current, and 1536 × 1536 matrix. Quality control was monitored through daily examinations of a phantom (Phanton) containing hydroxyapatite rods (HA) incorporated in an equivalent resin fabric (QRM, Moehrendorf, Germany). Daily device calibration was performed to obtain images with good technical quality; parameters were kept stable throughout the study period.

All measurements were obtained using the same scanner, and one biomedical specialist analyzed the data according to guidelines^21^. The quality of the scan image was categorized as follows: perfect (G1), showing a slight (G2); moderate (G3); or unacceptable (G4) degree of movement artifact^34^. As the presence of movement artifacts was unacceptable, for the distal tibia, only 11 exams were repeated due to movement during the exam (talking, coughing).

The following trabecular parameters were analyzed: Tb.vBMD, trabecular volumetric density (mg HA/cm^3^); Tb.N, number of trabeculae (1 mm, SD); Tb.Th, trabecular thickness (mm); Tb.Sp, trabecular separation (mm); and cortical parameters: Ct.vBMD, cortical volumetric density (mg HA/cm^3^); Ct.Th, mean cortical bone thickness (mm); and Ct.Po, cortical porosity.

In addition to standard morphological analysis, automatic segmentation software called “advanced cortical analysis” was used to determine cortical parameters^35, 36^.

The HR-pQCT precision measurement from our laboratory, expressed as the coefficient of variation, ranged from 0.25 to 1.16% for the density parameters at the tibia site^37^.

### Areal bone mineral density

Areal bone mineral density at the lumbar spine, femoral neck, and total hip were measured using DXA (Hologic QDR 4500, Inc. Bedford, MA, USA), and the results are expressed in g/cm^2^. The *T*-score was applied as a noninvasive tool for osteoporosis diagnosis and as an addendum to the original description by the WHO criteria definition threshold^30, 38^. The same DXA scanner was used for all single exams, and the results were evaluated by one biomedical specialist. The least significant changes (LSCs) for BMD measurement were 0.033 g/cm^2^ at the lumbar spine, 0.047 g/cm^2^ at the femoral neck, and 0.039 g/cm^2^ at the total hip.

### Statistical analysis

Statistical analysis was performed using STATA statistical software, version 12.0 (Stata Corp., College Station, USA). Continuous variables are expressed as medians and interquartile ranges or means ± standard deviations depending on the Kolmogorov–Smirnov test for normality. Continuous variables in the total sample were separated by sex and analyzed for differences with Student’s *t* test if they showed a parametric pattern or the Mann–Whitney test if they did not (as with insulin and RCP values). Linkert scales such as for alcohol consumption and degree of physical activity were also evaluated by the Mann–Whitney test. In the case of binomial variables, a Chi-squared test was performed, and Fisher’s adaptation was used as needed. The study’s response variable was the calcium score, as expressed by its integer value. This variable was grouped by sex, assumed nonnegative integer values, with an asymmetric distribution on the left. Variables related to HR-pQCT were standardized in *z* scores using the Formula: z = (observed value − sample mean)/sample standard deviation^39^. *T*-scores were used to evaluate bone density compared to normal values in a young adult and formed part of the regression model. In univariate analysis, variables with p < 0.20 were used in the regression model with a stepwise variable selection procedure. Analysis of association was performed using a generalized linear regression model with a Poisson probability distribution because of the nonparametric distribution of calcium content. In addition, a layered stratification approach was taken: (1) by sex; and (2) by anatomical site. Thus, there were four groups (radio men, tibia men, radio women and tibia women). All tests were two-tailed, and p values < 0.05 were considered statistically significant.

## Results

### Anthropometric and clinical characteristics

The baseline patient characteristics regarding anthropometric and clinical variables are shown in Table 1. The mean age was 79.5 ± 4.24 years without sex differences. Women represented the largest group (65%). In them was observed a higher percentage of non-white women compared to men. Waist circumference was similar in both groups. BMI, hypertension, and diabetes were significantly higher in women, and consequently, women had a higher prevalence of metabolic syndrome. Stroke and osteoporosis prevalence was also higher in women. In contrast, alcohol consumption was higher in men. Different grades of physical activity from low to high were similar in both groups. Although the women had more fall histories and worse BT scores, they had similar new fracture events when compared to men.

**Table 1:** Anthropometric and clinical characteristics of 256 older adults, separated by sex.

Variable	Total N = 256	Women N = 168	Men N = 88	p value
Age (years)	79.5 ± 4.2 (68–94)	79.7 ± 4.2 (70–94)	79 ± 4.3 (68–92)	0.200
Height (cm)	153.2 ± 8.8	149 ± 6.9	161.2 ± 7.5	< 0.001
Weight (kg)	67.4 ± 12	66.4 ± 12.2	69.3 ± 11.6	< 0.070
WC (cm)	96 ± 11.1	96.6 ± 11.5	94.8 ± 10.2	0.223
BMI (kg/cm^2^)	28.7 ± 4.9	29.9 ± 5.0	26 ± 4.2	< 0.001
**Race**				
White	75 (29.3%)	44 (26.2%)	31 (35.2%)	0.310
Non-White	181 (70.7%)	124 (73.8%)	57 (64.8%)	
SBP (mmHg)	158 ± 23 (105–220)	159 ± 23 (105–220)	154 ± 23 (105–217)	0.382
DBP (mmHg)	84 ± 12 (56–131)	84 ± 15 (60–196)	84 ± 13 (56–124)	0.560
Alcohol consumption*				
Current	4 (1.6%)	0 (0%)	4 (4.6%)	< 0.001
Last 3–4 years	2 (0.8%)	0 (0%)	2 (2.3%)	
Never	201(78.5%)	143 (85.1%)	58 (65.9%)	
Social	49 (19.1%)	25 (4.9%)	24 (27.3%)	
Smoking	24 (9.4%)	7 (8%)	17 (10.1%)	0.570
Degree of physical activity*				
Low	40 (15.6%)	28 (16.7%)	12 (13.6%)	0.199
Moderate	149 (58.2%)	102 (60.7%)	47 (53.4)	
High	67 (26.2%)	38 (22.6%)	29 (33%)	
Coffee Consumption	231 (90.2%)	156 (92.9%)	75 (85.2%)	0.051
DM	58 (22.7%)	42 (25%)	16 (18.2%)	0.210
Metabolic syndrome	103 (40.2%)	84 (50.6%)	19 (21.6%)	< 0.001
HT	192 (75%)	133 (79.2%)	59 (67.1%)	0.030
DLP	117 (45.7%)	89 (53%)	28 (31.8%)	0.001
Angina	1 (0.4%)	1 (0.6%)	0 (0%)	1.000
MI	8 (3.1%)	5 (3%)	3 (3.4%)	1.000
Stroke	20 (7.8%)	9 (5.4%)	11 (12.5%)	0.040
DVT	6 (2.3%)	5 (3%)	1 (1.1%)	0.660
BT score ≤ − 2.5	101 (39.5%)	81 (48.2%)	20 (22.7%)	< 0.001
New fracture	17 (6.6%)	14 (8.3%)	3 (3.4%)	0.130
Fall	76 (29.7%)	58 (34.5%)	18 (20.5%)	0.010

Continuous variables are represented as the mean ± standard deviation (*t* test); discontinuous variables are represented as the median and interquartile range (Mann–Whitney *U* test); binomial variables are represented as the number and percentage (Chi2 test); *Variables with Linkert scales were analyzed by the Mann–Whitney *U* test. *WC* waist circumference, *BMI* body mass index, *SBP* systolic blood pressure, *DBP* diastolic blood pressure, *DM* diabetes, *HT* hypertension, *DLP* dyslipidemia, *MI* myocardial infarction, *DVT* deep-vein thrombosis, *BT score* bone T score.

**Figure 2:**
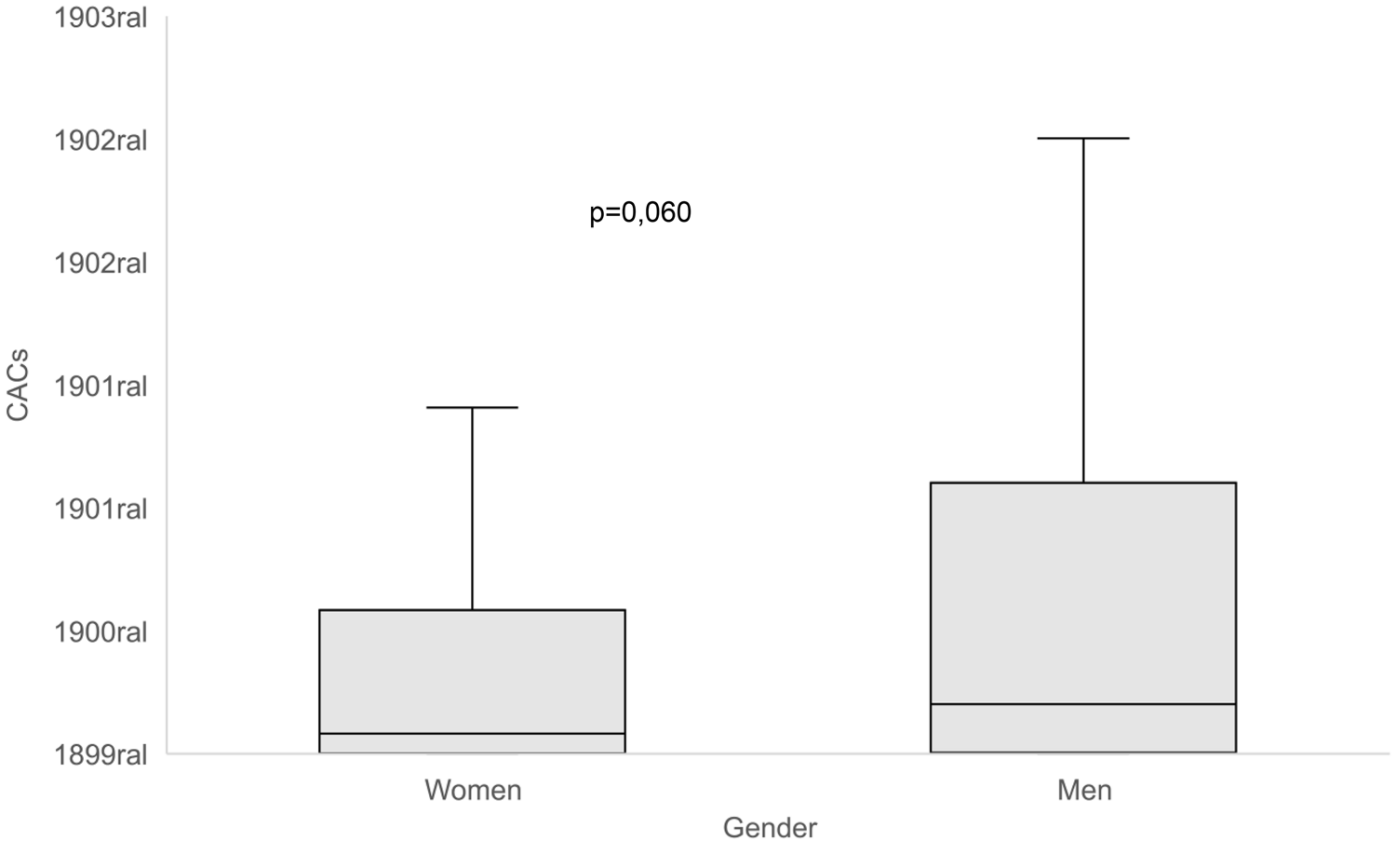
Coronary calcium content between the sexes. CACs values are expressed in a logarithmic display and sex comparisons made by Mann–Whitney test.

Regarding coronary calcium, median CAC values did not differ significantly between the groups (Fig. 2).

### Laboratory characteristics

Lipid indices (CT, HDL, and LDL) and phosphorus were higher in women; carbohydrate metabolism, as represented by glucose and insulin, was similar for both groups. Although plasma creatinine was different between the groups, renal function, as measured by clearance, was similar for men and women (Table 2).

**Table 2:** Laboratory characteristics of 256 older adults, separated by sex.

Variable	Total N = 256	Women N = 168	Men N = 88	p value
Blood glucose, mg/dL	102 ± 28	103 ± 30	101 ± 24	0.482
Total Cholesterol, mg/dL	207 ± 44	214 ± 45	192 ± 37	< 0.001
HDL, mg/dL	56 ± 15.16	59 ± 15	50 ± 13	< 0.001
LDL, mg/dL	126 ± 40	129 ± 43	119 ± 33	0.030
Triglycerides, mg/dL	132 ± 63	121 ± 63	121 ± 63	0.054
Creatinine, mg/dL	0.92 ± 0.28	0.83 ± 0.20	1.08 ± 0.32	< 0.001
ClCr mL/min/1.73 m^2^	75.71 ± 19	78.55 ± 33	74.73 ± 20	0.544
Alkaline phosphatase, U/L	76.92 ± 29	78.55 ± 33	73 ± 21	0.216
Phosphorus, mg/dL	3.34 ± 0.49	3.47 ± 0.46	3.08 ± 0.45	< 0.001
Calcium, mg/dL	9.68 ± 0.49	9.7 ± 0.51	9.63 ± 0.46	0.291
iPTH, pg/dL	56.79 ± 27	56.1 ± 22	58.09 ± 35	0.627
25(OH)D, ng/mL	23 ± 9	23 ± 9	24 ± 9	0.700
Insulin, mU/L*	10.90 [6.98–16.7]	11.8 [7.5–18.7]	9.05 [5.9–13.2]	< 0.001
CRP, mg/L	3.90 ± 9.57	4.03 ± 9.71	3.65 ± 9	0.071
CTX, ng/mL	0.27 ± 0.17	0.27 ± 0.17	0.27 ± 0.17	0.985
P1NP, ng/mL	39 ± 26	40 ± 28	37 ± 20	0.355

The mean ± standard deviation was adopted for all variables (*t* test), except for insulin* represented with median + interquartile range 25–75 (Mann–Whitney *U* test); *HDL* high-density lipoprotein, *LDL* low-density lipoprotein, *ClCr* estimated creatinine clearance, *iPTH* parathyroid hormone, *25OHD* 25-hydroxyvitamin D, *CRP* C-reactive protein, *CTX* collagen type I amino-terminal telopeptide, *P1NP* amino-terminal propeptide of type I collagen. p value compares female vs. male variables.

### Medication use

Table 3 shows that thiazide diuretics were the most used medication (and more frequently in women), followed by statins and beta-blockers. Regarding osteoporosis, specific treatment was used more by women than men: almost twice as much for calcium and 25(OH)D and with a higher proportion for bisphosphonate. Unfortunately, neither the specific kind of bisphosphonate nor the dosage of vitamin D was recorded.

**Table 3:** Absolute and relative frequencies of medications used in 256 older adults, separated by sex.

Variable	Total N = 256—%		Women N = 168—%		Men N = 88—%		p value
ACEI	89	34.8	58	34.5	31	35.2	0.910
Thiazide	109	42.6	81	48.2	28	31.8	0.012
Calcium blocker	47	18.4	35	20.8	12	13.6	0.150
Beta-blocker	53	20.7	42	25.0	11	12.5	0.010
Statin	83	32.4	64	38.1	19	21.6	0.007
ASA	72	28.1	45	26.8	27	30.7	0.510
Warfarin	4	1.6	3	1.8	1	1.1	1.000
Levothyroxine	31	12.1	26	15.5	5	5.7	0.020
Insulin use	17	6.6	11	6.6	6	6.8	0.940
Supplemental calcium	86	33.6	69	41.1	17	19.3	< 0.001
25(OH)D	111	43.4	91	54.2	20	22.7	< 0.001
Bisphosphonate	98	38.3	84	50.0	14	15.9	< 0.001
Glucocorticoid	4	1.6	3	18	1	1.1	1.000

*ACEI* angiotensin-converting enzyme inhibitor, *ASA* acetylsalicylic acid, *25*(*OH*)*D* 25-hydroxyvitamin D.

### Bone mineral density and bone microarchitecture based on CAC

The median CAC value was 54.5 (IQR 1–290.75) for the general population, with no difference observed between women [33 (IQR 0.25–236.5) and men (80.5 (IQR 1.25–450.25)], p = 0.06 (supplementary information).

In univariate analysis, total hip aBMD in both sexes and femoral neck aBMD in men were associated with lower CAC. Inversely, lumbar spine aBMD in both sex, and femoral neck aBMD in women, were associated with higher coronary calcification. Also, we found that *T*-score ≤ − 2.5 SD, was associated with higher coronary calcification in women; however, the association was the opposite in men.

Regarding HR-pQCT parameters, Ct.vBMD and Ct.Po in women at the tibia was negatively and positively associated with CAC, respectively. In men, more variables were associated with higher CAC, namely, Ct.vBMD, CT.Po, Tb.N, and Ct.Th. Overall, trabecular parameters were positively associated with CAC, with a 31–47% chance for each standard deviation of the *z* score. For the radius, only CT.Po had a positive association with CAC in women, and the other parameters followed an opposite trend. In men, Ct.vBMD, Ct.Th, and CT.Po were positively associated with CAC (Tables 4 and 5).

**Table 4:** Bone mineral density (DXA) and bone microarchitecture (HR-pQCT) for CAC status (*z* score) in women.

Variable	Exp β	95% CI		p value
**DXA**				
aBMD lumbar spine (g/cm^2^)	1.1159	1.1003	1.1317	< 0.001
aBMD femoral neck (g/cm^2^)	1.1020	1.0863	1.1180	< 0.001
aBMD total hip (g/cm^2^)	0.9716	0.9571	0.9862	< 0.001
BT score ≤ − 2.5 SD	1.1118	1.0891	1.1350	< 0.001
**HR-pQCT**				
Tibia				
Tb.vBMD (mg HA/cm^3^)	1.3139	1.2984	1.3296	< 0.001
Ct.vBMD (mg HA/cm^3^)	0.7629	0.7521	0.7738	< 0.001
Tb.N (1/mm)	1.0620	1.0501	1.0740	< 0.001
Tb.Th (mm)	1.3100	1.2973	1.3228	< 0.001
Tb.Sp (mm)	0.8662	0.8566	0.8759	< 0.001
Ct.Th (mm)	1.0112	0.9990	1.0236	0.072
Ct.Po (1)	1.1743	1.1614	1.1872	< 0.001
Radius				
Tb.vBMD (mg HA/cm^3^)	1.2219	1.2070	1.2371	< 0.001
Ct.vBMD (mg HA/cm^3^)	1.1900	1.1757	1.2045	< 0.001
Tb.N (1/mm)	1.1025	1.0904	1.1148	< 0.001
Tb.Th (mm)	1.0734	1.0639	1.0831	< 0.001
Tb.Sp (mm)	0.8708	0.8608	0.8809	< 0.001
Ct.Th (mm)	1.2539	1.2391	1.2689	< 0.001
Ct.Po (1)	1.0601	1.0539	1.0664	< 0.001

*Exp β* equation coefficient, *aBMD* areal bone mineral density, *BT score* bone *T*-score, *SD* standard deviation, *Tb.vBMD* trabecular volumetric density, *Ct.vBMD* cortical volumetric density, *Tb.N* trabeculae number, 1 mm, *Tb.Th* trabecular thickness, *Tb.Sp* separation between trabeculae, *Ct.Th* cortical thickness, *CT.Po* cortical porosity, *CI* confidence interval.

**Table 5:** Bone mineral density (DXA) and bone microarchitecture (HR-pQCT) for CAC status (*z* score) in men.

Variable	Exp β	CI	95%	p value
**DXA**				
aBMD lumbar spine (g/cm^2^)	1.0006	0.9881	1.0132	0.928
aBMD femoral neck (g/cm^2^)	0.9113	0.9010	0.9217	< 0.001
aBMD total hip (g/cm^2^)	0.9506	0.9392	0.9621	< 0.001
BT score ≤ − 2.5 SD	0.7281	0.7088	0.7479	< 0.001
**HR-pQCT**				
Tibia				
Tb.vBMD (mg HA/cm^3^)	1.0309	1.0191	1.0428	< 0.001
Ct.vBMD (mg HA/cm^3^)	0.6745	0.6643	0.6848	< 0.001
Tb.N (1/mm)	0.7417	0.7320	0.7516	< 0.001
Tb.Th (mm)	1.4704	1.4522	1.4888	< 0.001
Tb.Sp (mm)	1.4147	1.3821	1.4480	< 0.001
Ct.Th (mm)	0.9184	0.9087	0.9283	< 0.001
Ct.Po (1)	1.3852	1.3733	1.3972	< 0.001
Radius				
Tb.vBMD (mg HA/cm^3^)	1.2304	1.2163	1.2447	< 0.001
Ct.vBMD (mg HA/cm^3^)	0.9114	0.9013	0.9216	< 0.001
Tb.N (1/mm)	1.2857	1.2672	1.3044	< 0.001
Tb.Th (mm)	1.1166	1.1009	1.1326	< 0.001
Tb.Sp (mm)	0.5924	0.5754	0.6098	< 0.001
Ct.Th (mm)	0.9264	0.9175	0.9355	< 0.001
Ct.Po (1)	1.3613	1.3302	1.3931	< 0.001

*Exp β* equation coefficient, *aBMD* areal bone mineral density, *BT score* bone *T*-score, *SD* standard deviation, *Tb.vBMD* trabecular volumetric density, *Ct.vBMD* cortical volumetric density, *Tb.N* average number of trabeculae, 1 mm, *Tb.Th* trabecular thickness, *Tb.Sp* separation between trabeculae, *Ct.Th* cortical thickness, *CT.Po* cortical porosity, *CI* confidence interval.

Multivariate analysis was adjusted for CAC and sex, the dependent variables were DXA and HR-pQCT parameters. Surprisingly, some tibia and radius DXA/HR-pQCT results in multivariate analysis were different between the sexes (Table 6), with paradoxical results, as described below.

**Table 6:** Regression models for calcium content using bone mineral density (DXA) and HR-pQCT parameters at the tibia and radius stratified for sex.

Variable	Exp β	CI	95%	p
**Tibia**				
Women				
BT score ≤ − 2.5 SD	1.03	1.00	1.05	< 0.001
Ct.Po z (1)	1.12	1.10	1.13	< 0.001
Ct.vBMD z mg HA/cm^3^	0.79	0.78	0.81	< 0.001
Men				
BT score ≤ − 2.5 SD	0.75	0.73	0.77	< 0.001
Ct.Po z (1)	1.44	1.42	1.46	< 0.001
Ct.vBMD z mg HA/cm^3^	1.10	1.07	1.13	< 0.001
**Radius**				
Women				
BT score ≤ − 2.5 SD	1.20	1.18	1.23	< 0.001
Ct.Po z (1)	1.07	1.07	1.08	< 0.001
Ct.vBMD z mg HA/cm^3^	1.22	1.20	1.24	< 0.001
Men				
BT score ≤ − 2.5 SD	0.73	0.71	0.75	< 0.001
Ct.Po z (1)	1.33	1.30	1.37	< 0.001
Ct.vBMD z mg HA/cm^3^	0.98	0.88	0.99	0.012

*Exp β* equation coefficient, *CI* confidence interval, *SD* standard deviation, *BT score* bone *T*-score, *Ct. Po* cortical porosity, *z* *z* score, *Ct.vBMD* cortical volumetric density.

### Tibia measurements

In women, regarding DXA results, a greater quantity of CACs was associated with BT score ≤ − 2.5 SD. Concerning HR-pQCT, CT.Po showed a positive relationship. In contrast, Ct.vBMD had an inverse relation of 21% for each unit in the standard deviation of the *z* score.

In men, BT score ≤ − 2.5 SD was significantly associated with lower CACs. Concerning HR-pQCT parameters, only CT.Po was associated with higher CAC, with a positive 44% chance for a unit in the calcium score, Ct.vBMD presented a coefficient opposite to what was expected compared to women.

### Radius measurements

In women, BT score ≤ − 2.5 SD was also significantly associated with higher CACs. About HR-pQCT parameters, CT.Po and Ct.vBMD were associated with greater coronary calcification.

In men, vascular calcification was not related to osteoporosis based only BT score ≤ − 2.5 SD. Concerning CT.Po, it was congruent not only with the radius measurements in women but also with the tibial measurements. However, in men, Ct.vBMD showed an inverse relationship with coronary calcification (Table 6).

## Discussion

In this cross-sectional study involving healthy older adults, we observed an association between CAC and HR-pQCT parameters. Our main findings suggest an inverse relationship between calcium content in the coronary arteries and bone microarchitecture.

Previous studies have revealed a relationship between CAC and bone density. Using DXA and multidetector tomography in postmenopausal women, Lee et al.^40^ reported an associated odds ratio of 2.79, 95% CI 1.05–6.90) for osteoporosis, with a CAC score above 100 and higher odds of multivessel disease (8.91, 95% CI 1.93–41.22), as adjusted for cardiovascular risk factors and age. Nonetheless, that study did not address intake of bone metabolism drugs.

Later, a large cohort study with an 8-year follow-up^41^ found increased mortality risk and CAC related to BMD. The study used the trabecular thoracic spine as a reference for BMD levels, and after stratifying CAC into four categories, the observed association was more robust in postmenopausal women than men. Interestingly, the authors also explored ethnicity and found a weaker link for Asians in the CAC 1–100 category. We were unable to investigate this link because our population was not ethnically diverse.

Cortical and trabecular bone loss exhibited different behaviors in renal patients. Costa et al.^42^ used vertebral tomography and observed that cortical bone loss in mid-term follow-up (24 months), unlike trabecular bone loss, was not associated with aortic calcification. One of the hypotheses to explain this lack of association was that cortical bone has lower porosity and turnover rates than trabecular bone. The study found no effect of bone-affecting medications (diuretics, calcium base phosphate binders, and calcitriol).

Another study evaluated the relationship between BMD (assessed by HR-pQCT) and ischemic heart disease (myocardial infarction and angina)^43^. Interestingly, lower cortical volumetric BMD in the distal radius but not in the tibia was found; the study did not estimate coronary calcium content or medications, which can modify bone turnover.

Other studies, however, showed inverse results between vessel calcification and BMD. Aoyagi et al.^44^ evaluated aortic calcification contrasting with different skeletal sites (calcaneus, proximal and distal radius) and failed to observe a relationship in women. Their rationale revolved around hormonal imbalances and a lack of knowledge about vitamin D intake.

To the best of our knowledge, only one study in the literature has compared these two biological parameters using highly accurate imaging methods. However, this study was performed in a specific population with chronic kidney disease on dialysis, in whom lower trabecular and cortical parameters were associated with higher coronary calcification^24^.

Our results showed that higher CT.Po in both sexes and lower Ct.vBMD in women were associated with higher CACs. Furthermore, BT score ≤ − 2.5 SD, as evaluated by DXA, was associated with higher CACs in women.

Regarding HR-pQCT analysis, changes in cortical bone parameters were more greatly influenced by age and sex^45, 46^. Mc Donald et al.^47^ showed a decline in Ct. BMD with age in both women and men (14% and 17%, respectively), and the increase in CT.Po was three times higher in women than in men. Whitmarsh et al.^48^ observed a significant decrease in Tb. BMD (38.1%) and Ct.Th (13%) using micro-CT on femoral head specimens from women undergoing hemiarthroplasty from the 9th to the 10th decade of life. The difference between the sexes likely involves the influence of testosterone supporting periosteal bone expansion and estrogen preventing bone loss^49^. This might explain why our findings were more evident in women.

Moreover, there is evidence of a different response to exogenous and endogenous factors, in addition to the metabolic and structural differences between cortical and trabecular tissue^50^. A study analyzing HR-pQCT reference curves in 450 healthy women aged 20–85 years observed an age-related increase in CT.Po in women, though trabecular parameters remained relatively stable^35^.

A hypothesis that might explain this antagonistic pattern is the existence of an intracortical remodeling transition area that increases porosity by the process of trabecularization of the internal cortex, which may cause underestimation in the measurement of cortical bone and overestimation in that of trabecular tissue^51^. This phenomenon is subject to ex vivo analysis using computed microtomography (μCT), HR-pQCT images from cadaveric specimens, or electron microscopy^52^. A study of postmortem femur specimens obtained from 24 white women aged 29–99 years (mean age 69 years) suggested that cortical thinning occurs owing to the coalescence of cortical pores rather than endocortical expansion^53^.

This behavior is likely amplified by interaction of the similar pathophysiological processes of the two illnesses. Because of the high accuracy of both methods in diagnosis and risk stratification, it may be relevant to continue investigating this association to obtain new prevention and treatment strategies that involve both pathologies, especially for females.

Furthermore, our results reinforce knowledge of the relationship between VC and decreased bone remodeling and have important clinical implications in the design of more specific diagnostic and treatment strategies for both pathologies.

We recognize the limitations of our study, as a cross-sectional design does not allow for inference of cause and effect in the association. The sample size might be considered small regarding the initial referential study, which confers less statistical power. The small number of participants in the group of men may explain those results, and the differences in bone-health medications were lower in this group.

In conclusion, we observed that cortical bone impairment at the tibia and radius, as assessed by HR-pQCT, is associated with increased coronary calcification in older people from the São Paulo Ageing and Health Study cohort. Otherwise, bone mineral density by DXA showed the opposite trend when related to coronary calcification depending on sex.

## Supplementary Information

Supplementary Table 1.
