# Supplementary material for: Coronary calcification and bone microarchitecture by high-resolution peripheral quantitative computed tomography from the São Paulo Ageing and Health (SPAH) Study
Source: Sci Rep. 2022 Mar 28;12:5282. doi: 10.1038/s41598-022-08839-0 (PMC8960801; doi:10.1038/s41598-022-08839-0)
Supplement: Supplementary file 1 — Supplementary Table 1. [file 41598_2022_8839_MOESM1_ESM.docx]

**Coronary calcification and bone microarchitecture by high-resolution peripheral quantitative computed tomography from São Paulo Ageing & Health (SPAH) Study**

Luis Fernando Escobar Guzman, Neuza Helena Moreira Lopes, Georgea H. Fernandes Torres, Liliam Takayama, Solange de Sousa Andrade, José Ramón Lanz-Luces, Rosa Maria R. Pereira, Carlos Eduardo Rochitte

**Supplementary information**

Coronary arterial calcification (CAC), bone mineral density (BMD) and HR-pQCT variables of 256 elderly individuals, separated by sex

|  | | | | |
| --- | --- | --- | --- | --- |
| \| Variable \|  \|  \|  \|  \| \| --- \| --- \| --- \| --- \| --- \| | **Total** | **Women** | **Men** | **P** |
| CACs, n | 256  283.95 (528.5)  54.50 [1-290.75] | 168  214.17 (388)  33 [0.25-236.5] | 88  417.16 (708.7)  80.5 [1.25-450.25] | 0.065 |
| aBMD lumbar spine,g/cm^2^, n | 251  0.94 (0.19)  0.92 [0.80-1.06] | 163  0.88 (0.17)  0.87 [0.75-0.97] | 88  1.05 (0.19)  1.02 [0.93-1.17] | <0.001 |
| aBMD a femoral neck, g/cm^2^, n | 253  0.68 (0.13)  0.67 [0.59-0.77] | 166  0.64 (0.11)  0.64 [0.56-0.72] | 87  0.76 (0.14)  0.75 [0.65-0.84] | <0.001 |
| aBMD total femur, g/cm^2^, n | 253  0.83 (0.15)  0.82 [0.73-0.94] | 166  0.78 (0.13)  0.78 [0.7-0.86] | 87  0.93 (0.15)  0.94 [0.8-1.06] | <0.001 |
| Tibia Tb.vBMD, mg HA/cm^3^, n | 254  135.24 (39.9)  132.45 [108-163] | 167  123.77 (36.65)  122.9 [101.6-151.3] | 87  157.27 (36.97)  155.7 [127.7-176] | <0.001 |
| Tibia Ct.vBMD, mg HA/cm3, n | 254  820.34 (70.53)  830.9 [776-865.7] | 167  805.17 (68.78)  817.4 [767.9-851.2] | 87  849.45 (64.8)  817.4 [767.9-851] | <0.001 |
| Tibia Tb.N, 1/mm, n | 254  1.53 (0.43)  1.51 [1.25-1.82] | 167  1.41 (0.42)  1.41 [1.16-1.69] | 87  1.76 (0.37)  1.70 [1.49-2,03] | <0.001 |
| Tibia Tb.Th, mm, n | 254  0.07 (0.01)  0.59 [0.48-0.73] | 167  0.92 (0.28)  0.92 [0.71-1.13] | 87  0.08 (0.01)  0.07 [0.06-0.08] | 0.83 |
| Tibia Tb.Sp, mm, n | 254  0.65 (0.29)  0.59 [0.48-0.73] | 167  0.72 (0.33)  0.64 [0.51-0.79] | 87  0.52 (0,13)  0.52 [0.41-0.59] | <0.001 |
| Tibia Ct.Th, mm, n | 254  1.01 (0.32)  1.03 [0.77-1,24] | 167  0.92 (0.28)  0.92 [0.71-1.13] | 87  1.19 (0.32)  1.21 [0.96-1.41] | <0.001 |
| Tibia Ct.Po (1), n | 253  0.08 (0.03)  0.08 [0.06-0.09] | 166  0.08 (0.03)  0.08 [0.06-0.10] | 87  0.08 (0.03)  0.07 [0.06-0.09] | 0.49 |

Legend - Mean (SD): standard deviation; median [IQR]: interquartile range; CACs: Coronary calcium score; aBMD: Areal bone mineral density; Tb.vBMD: Trabecular volumetric density; Ct.vBMD: Cortical volumetric density; Tb.N: number of trabeculae; Tb.Th: trabecular thickness; Tb.Sp: separation between trabeculae; Ct.Th: cortical thickness; Ct.Po cortical porosity.
